# Evolution, development, and plasticity of the human brain: from molecules to bones

**DOI:** 10.3389/fnhum.2013.00707

**Published:** 2013-10-30

**Authors:** Branka Hrvoj-Mihic, Thibault Bienvenu, Lisa Stefanacci, Alysson R. Muotri, Katerina Semendeferi

**Affiliations:** ^1^Department of Anthropology, University of California at San DiegoLa Jolla, CA, USA; ^2^Department of Pediatrics/Rady Children’s Hospital San DiegoDepartment of Cellular and Molecular MedicineStem Cell Program, University of California at San Diego, School of MedicineLa Jolla, CA, USA.; ^3^Neuroscience Graduate Program, University of California at San DiegoLa Jolla, CA, USA

**Keywords:** pyramidal neurons, plasticity, neuropsin, brain evolution, development, amygdala, endocast, human evolution

## Abstract

Neuroanatomical, molecular, and paleontological evidence is examined in light of human brain evolution. The brain of extant humans differs from the brains of other primates in its overall size and organization, and differences in size and organization of specific cortical areas and subcortical structures implicated into complex cognition and social and emotional processing. The human brain is also characterized by functional lateralizations, reflecting specializations of the cerebral hemispheres in humans for different types of processing, facilitating fast and reliable communication between neural cells in an enlarged brain. The features observed in the adult brain reflect human-specific patterns of brain development. Compared to the brains of other primates, the human brain takes longer to mature, promoting an extended period for establishing cortical microcircuitry and its modifications. Together, these features may underlie the prolonged period of learning and acquisition of technical and social skills necessary for survival, creating a unique cognitive and behavioral niche typical of our species. The neuroanatomical findings are in concordance with molecular analyses, which suggest a trend toward heterochrony in the expression of genes implicated in different functions. These include synaptogenesis, neuronal maturation, and plasticity in humans, mutations in genes implicated in neurite outgrowth and plasticity, and an increased role of regulatory mechanisms, potentially promoting fast modification of neuronal morphologies in response to new computational demands. At the same time, endocranial casts of fossil hominins provide an insight into the timing of the emergence of uniquely human features in the course of evolution. We conclude by proposing several ways of combining comparative neuroanatomy, molecular biology and insights gained from fossil endocasts in future research.

## INTRODUCTION

The search for the evolutionary emergence of neural features underlying human cognitive and behavioral specializations represents a persistent field of inquiry spanning several disciplines. From comparative neuroanatomy through molecular biology and paleoanthropological reconstructions, years of research have yielded numerous insights into features unique to the human brain, their morphological correlates, evolutionary pathways, and context of their appearance. Compared to other primates, extant humans are unique in the nature of their sociality, ecological adaptations, and, most importantly, in a complete reliance on culture as the extrasomatic, transgenerationally transmitted behavioral adaptation (Alexander, 1989; Kaplan et al., 2000; Hill et al., 2009). Throughout the evolution of the genus *Homo*, the fossil record demonstrates an increase in brain size and appearance of cortical asymmetries suggestive of functional lateralization (Falk, 1987; Holloway et al., 2004). At the same time, comparative neuroanatomical studies suggest that, in addition to an increase in size, human brain evolution was characterized by selective enlargement and reorganization of specific cortical areas (Semendeferi and Damasio, 2000; Semendeferi et al., 2001, 2011) and subcortical structures (Barger et al., 2007, 2012), potentially promoting information processing unique to our species. In parallel, human life history is characterized by an extended period of offspring dependency compared to chimpanzees, delayed onset of reproductive maturation, and long post-reproductive life-span (Bogin and Smith, 1996; Flinn, 2005; Hawkes, 2006), enabling prolonged cognitive maturation, acquisition of skills necessary for survival, and their transmission across generations.

The importance of complex morphological structures and flexible behaviors – allowing for novel responses to newly encountered selective pressures – was proposed as the key adaptation of the hominin lineage (Potts, 1998). In this sense, variability selection approached human evolution from a perspective different from fluctuating selection and developmental plasticity; it emphasized the evolutionary emergence of traits capable of providing selective advantage to hominins in unstable conditions, without invoking changes in the reaction norm or the need for genetic polymorphisms (Potts, 1998). Among these traits, expansion of the brain and behavioral complexity emerged as the key features carrying a selective advantage during the course of human evolution.

Behavioral variability, together with a more general cognitive complexity, has been typically considered in the context of overall encephalization. However, the relationship between the brain size of fossil hominins and their behavioral complexity inferred from the archaeological remains is neither simple nor straightforward (McBrearty and Brooks, 2000; Teyssandier, 2008). Whereas the first wave of increase in brain size early in the Pleistocene coincides with the appearance of first bifacial tools, the relationship becomes less clear later in human evolution, especially when assessing cognitive capacities of early modern *H. sapiens*. Although it has been proposed that novel tool technologies, new food procurement strategies, and the emergence of representational art appeared suddenly and concurrently at 50–40 kya (Klein, 2000; Bar-Yosef, 2002), recent reports provide evidence that aspects of behavioral modernity may have already been present much earlier than that (McBrearty and Brooks, 2000; Brown et al., 2012). At the same time, anatomically modern humans were characterized by only a modest increase in the brain size compared to their predecessors (Ruff et al., 1997) leading some to suggest that the emergence of behavioral modernity may have been accompanied by subtle changes in cortical organization that cannot be inferred from the fossil record (Klein, 2000). The debate on the origin of behavioral modernity aside, changes in brain size are accompanied by numerous modifications in organization and connectivity. In the case of the neocortex, an expansion in cortical size tends to be accompanied by changes including absolute or relative size of cortical fields, enlargement of areas devoted to processing relevant sensory inputs, and changes in the amount of areas devoted to processing specific types of stimuli (Krubitzer and Kaas, 2005). Cortical expansion is often accompanied by an increase in modularity and a reduction in long axonal projections, thus decreasing the distance between neurons subserving the same set of information processing (Kaas, 2000).

A growing body of research suggests that neocortical pyramidal neurons – the basic units of cortical microcircuitry (DeFelipe et al., 2002) – display variations in homologous areas across primates, possibly underlying differences in cognitive potentials across taxa (Elston et al., 2006). As such, natural selection may have acted specifically on the morphology and organization of neurons, favoring a particular type of information processing in a given species (Kaas, 2000). When compared across primates, pyramidal neurons in humans tend to display more complex morphologies (Elston, 2003) that are capable of sampling from larger inputs and of participating in more extensive cortical networks (Jacobs and Scheibel, 2002). In all primates examined to date, pyramidal neurons are characterized by extensive morphological changes during post-natal maturation and remodeling throughout life, potentially underlying flexible behavioral responses typical of all primates. Pyramidal neurons in the human neocortex display a prolonged period of development compared to other primates (Cupp and Uemura, 1980; Petanjek et al., 2008, 2011), especially in the cortical areas characterized by expansion during human evolution, including selected areas in the prefronal cortex (PFC). Similar developmental differences can be observed in gene expression studies, with delayed peak activity of genes involved in synaptogenesis and neuronal plasticity in humans compared to chimpanzees and macaques (Liu et al., 2012). At the same time, certain genes implicated in neuronal plasticity display mutations unique to humans (Lu et al., 2007, 2009), potentially suggesting differences in regulation of these processes between humans and non-human primates.

Even though insights into the microstructure of the cortex gained from comparative neuroanatomical studies cannot be directly compared with the fossil crania, certain features of human brain development and cortical organization allow for a synthesis of paleontological, neuroanatomical, and molecular evidence in reconstructing human brain evolution. In this review, we will combine these lines of research to examine plasticity in the human brain from an evolutionary perspective. We will specifically address maturation, cortical asymmetries, and lifelong changes in human neocortical pyramidal neurons, molecular aspects underlying neocortical plasticity, and a potential time-frame for the evolution of increased plasticity in the human brain based on the insights gained from fossil endocasts. Where possible, we will refer to the evolution of subcortical structures, especially in relation to social and ecological adaptations unique to our species. Several specificities of the human brain, including its size, development, and hemispheric dominance can be examined in extant primates, traced through the course of human evolution, considered in the context of developmental patterns unique to the human brain, and supplemented by insights from molecular studies.

## HUMAN BRAIN EVOLUTION: INSIGHTS FROM THE NEURONAL PHENOTYPES

During the course of human evolution, the brain underwent an increase in its overall size (Falk et al., 2000; Holloway et al., 2004), in the relative size of some of its gross components (Finlay and Darlington, 1995; Semendeferi and Damasio, 2000), and a selective enlargement of specific cortical areas and subcortical nuclei (Semendeferi et al., 2001; Barger et al., 2007). Along with changes in size came subtle modifications in organization, indicating possibly significant alterations in microcircuitry at the cellular level (Semendeferi et al., 2011; Barger et al., 2012). From an anatomical perspective, morphological characteristics of a particular cortical region reflect the number, size, and distribution of neurons within that region (DeFelipe et al., 2002). Thus, an analysis of properties and organization of neurons in homologous areas across species forms the basis for examining cortical organization from an evolutionary point of view (Kaas, 2000). Increasingly there is interest in the level of individual neurons and how they vary across functionally different cortical areas, across species, and how they change across the lifetime (Jacobs et al., 2001; Sherwood et al., 2003a; Bianchi et al., 2012). Analyses at the neuronal level enable the development of testable hypotheses linking the morphology of information processing units and their function. They can also provide insights into plastic responses to environmental circumstances across different cortical areas, the limits of the plasticity, and possible differences in the nature or extent of plasticity across species.

**FIGURE 1:**
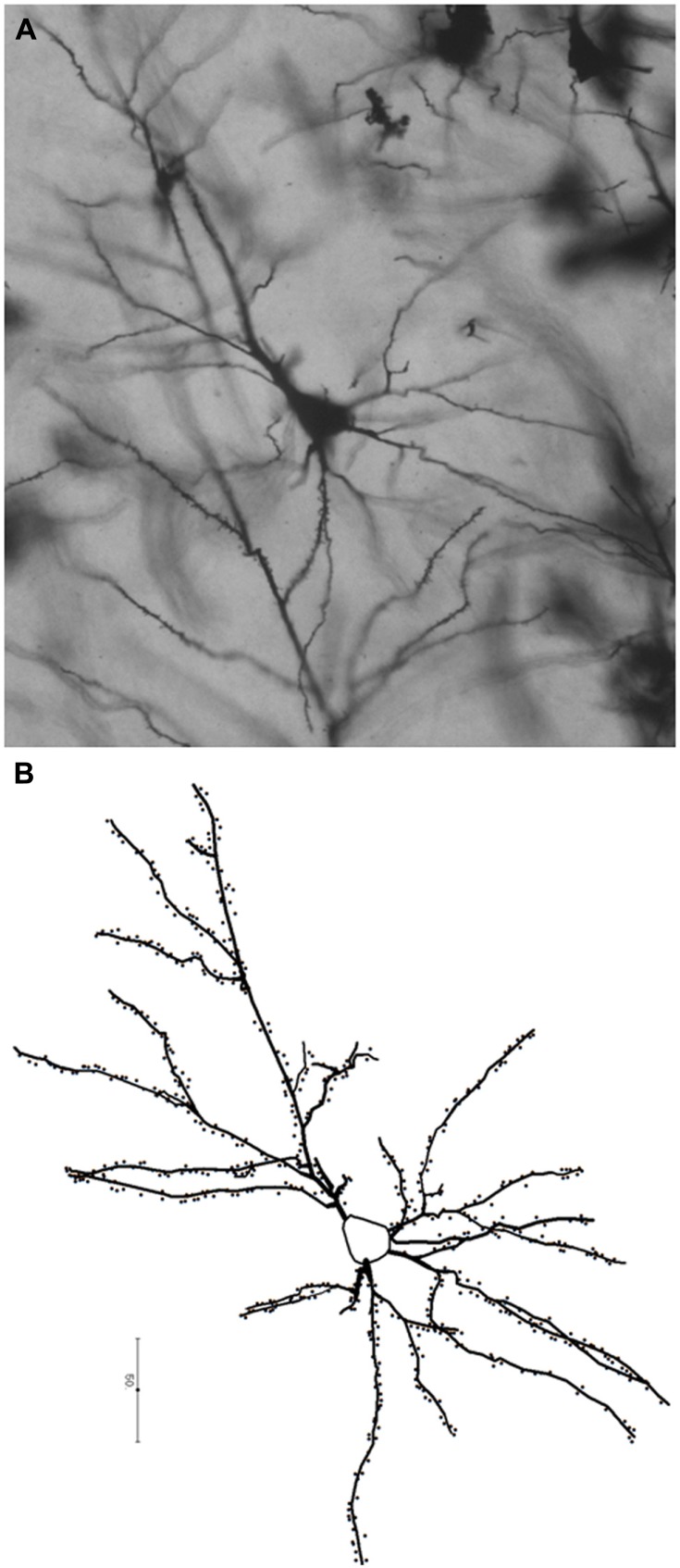
**Photomicrograph (A) and a schematic representation **(B)** of a pyramidal neuron from the human prefrontal cortex (BA 10) processed with the Golgi–Kopsch method.** Scalebar in **(B)** is in microns.

At the cellular level, the neocortex consists of excitatory pyramidal and spiny stellate neurons, and of various classes of inhibitory neurons (Nieuwenhuys, 1994; Hof and Sherwood, 2007; DeFelipe et al., 2013). Despite this cellular diversity, neocortical pyramidal neurons constitute the principal class of neurons in the cortex, accounting for 70–85% of all cortical neurons (DeFelipe and Fariñas, 1992) and have been the target of a considerable number of developmental, comparative, and evolutionary studies.****Pyramidal neurons form the basic units of cortical microcircuitry, determining the pattern of inputs and outputs into a particular cortical area (DeFelipe et al., 2002). In this review, we focus specifically on this morphological class of neurons. Pyramidal neurons are typically characterized by a pyramidal- or ovoid-shaped soma, the presence of one apical dendrite directed toward the pial surface, several basal dendrites emerging from sides of the soma, an axon emerging from the base of the cell body or from the proximal parts of basal dendrites, and the presence of spines representing sites of excitatory inputs onto dendrites (**Figure 1**; DeFelipe and Fariñas, 1992; Nieuwenhuys, 1994; Spruston, 2008).

In the cortex of adult primates – more specifically, macaques, chimpanzees, and humans – pyramidal neurons vary across cortical areas in the length of dendrites, branching complexity, and in number and density of dendritic spines (Cupp and Uemura, 1980; Jacobs et al., 1997, 2001; Elston, 2007; Bianchi et al., 2012). Pyramidal neurons in the primate neocortex also tend to display two trends: an increase in complexity in relatively larger cortical regions, and an increase in complexity from primary to higher-order sensory processing areas (Elston, 2003; Elston et al., 2006). In all three species, pyramidal neurons in the prefrontal cortex (PFC) tend to be longer, more branched, and more spinous compared to primary sensory areas. Across species, pyramidal neurons in the human cortex typically emerge as morphologically the most complex when compared to homologous areas of other primates, with the difference being particularly prominent in PFC pyramidal neurons (Elston, 2000; Elston et al., 2006; Bianchi et al., 2012). The prefrontal cortex comprises several cytoarchitectonically defined areas, and many of them, especially the ones within the dorsolateral PFC, are involved in complex cognitive tasks and executive functions in primates (Goldman-Rakic, 1987; Barbas, 1995). During the evolution of the human lineage, parts of the prefrontal regions, notably the frontopolar part, underwent an increase in size (Semendeferi et al., 2001) and changes in neuronal organization (Semendeferi et al., 2011), potentially indicating localized microanatomical changes related to cognitive complexity typical to humans. Analyses of pyramidal neurons in macaque, chimpanzee, and human cortex suggest that an increased complexity of PFC neurons in all species may reflect a trend toward emphasis on executive functions shared by Old World monkeys, apes, and humans (Elston, 2000; Elston et al., 2009; Bianchi et al., 2012), while the integrative role of PFC and its complex behaviors became even further emphasized in humans.

Reorganization observed in the human neocortex has been argued to parallel reorganization in some subcortical structures (Barton and Harvey, 2000). Among those, the amygdala emerges as critical in mediating social and emotional behavior in both human and non-human primates. While subcortical structures are generally considered to be conserved during primate evolution, the amygdala is anatomically connected with many neural systems that are differentially expanded in humans, such as parts of the prefrontal cortex and the temporal lobe (Stefanacci et al., 1996; Semendeferi and Damasio, 2000; Semendeferi et al., 2001; Stefanacci and Amaral, 2002). Amygdala connections with the prefrontal cortex are an important component of the social brain circuitry. Between 85 and 95% of neurons in the basal nucleus of the amygdala that project to the prefrontal cortex are pyramidal cells immunoreactive for the excitatory amino acids glutamate or aspartate (McDonald, 1996), suggesting the excitatory nature of amygdaloid inputs into the PFC.

When compared to the other members of the family Hominidae, namely chimpanzees, gorillas, bonobos, and orangutans, the amygdala in humans displays disproportional enlargement in the lateral nucleus (Barger et al., 2007, 2012) – both in terms of volume and number of neurons – suggesting a reorganization of the amygdaloid complex and an emphasis on functions processed in the lateral nucleus. This may reflect the primary connective relationship between the lateral nucleus and the temporal lobe (Stefanacci et al., 1996; Stefanacci and Amaral, 2002), which has also expanded over the course of evolution (Semendeferi and Damasio, 2000). The lateral nucleus also receives the majority of cortical sensory information directed to the amygdala (Stefanacci and Amaral, 2000, 2002; Ghashghaei and Barbas, 2002; Barbas et al., 2011), and it has been suggested that its expansion in humans may represent a heightened need to process more expansive and complex social stimuli and interactions (Barger et al., 2012). In addition, it has been argued that several features that set human cultures apart from behavioral traditions of non-human primates include socially shared regulation of behavior and emotional reinforcement of cultural rules (Hill et al., 2009), both of which may emphasize processing in central executive cortical regions as well as in the amygdala.

The neurons in the amygdala are morphologically suited to provide the foundation for their functional connectivity with numerous other brain regions. The morphology of neurons in the adult amygdala was described through Golgi studies dating back to 1928 (Gurdjian, studies in the rat). Spiny, pyramidal-like neurons and spine-sparse stellate neurons were first described by Hall (1972) in the cat and Braak and Braak (1983) carried out the first Golgi study in the human amygdala. The morphology of neurons in the basolateral complex (lateral, basal, and accessory basal nuclei) has been especially well described. In the adult amygdala, spiny, pyramidal-type neurons, and spine-sparse or aspiny stellate neurons have been identified in the basolateral complex of all species studied to date, including rats, cats, monkeys, and humans (for review see McDonald, 1992). These neurons are very similar to their counterparts in the cerebral cortex. Each of the other amygdaloid nuclei also contain at least one type of projection neuron that is spine dense and one type of spine-sparse neuron that appears to be a local circuit neuron (McDonald, 1992).

Most of the spiny neurons in the basolateral complex have a pyramid-shaped soma with a main dendrite that is longer than the other basal processes, like cortical pyramidal neurons. Unlike cortical pyramidal neurons, however, the basolateral neurons do not exhibit a preferential orientation. The soma and proximal part of the dendrites are smooth while more distal regions are characterized by pedunculated spines. The dendrites generally do not extend beyond nuclear boundaries or into the adjacent white matter, but axons have been observed to cross nuclear boundaries to join fiber bundles. This suggests that these represent projection neurons. An effective marker that can be used to identify pyramidal neurons in the basolateral complex is calcium/calmodulin-dependent protein kinase II (CaMKII), which has a critical role in long-term potentiation. When CaMKII was analyzed for neuronal localization in the basolateral nucleus of rats, virtually every pyramidal neuron appeared to be CaMKII-positive while non-pyramidal neurons were unstained (McDonald et al., 2002). Indeed, decades of studies in rats have demonstrated the importance of long-term potentiation in the amygdala for emotional learning and memory (Clugnet and LeDoux, 1990; Maren, 1999). Thus, the neurons in the basolateral complex of the amygdala are equipped to mediate the need for behavioral modifications encountered throughout life.

### DENDRITIC ASYMMETRIES IN THE HUMAN CORTEX

Cerebral hemispheres in humans, more so than the hemispheres of other primates, are specialized for different types of information processing (Gazzaniga, 2000; Sun and Walsh, 2006). Although communication between the hemispheres still remains important in humans (Gazzaniga, 2000), certain functions are preferentially processed in one hemisphere over the other. In processing of spatial and face recognition, the right hemisphere exerts dominance over the left hemisphere, whereas language processing tends to be subserved by the areas located in the left hemisphere (Geschwind, 1978; Geschwind and Miller, 2001). Asymmetries observed at the gross level in the human cortex represent structural correlates of functional lateralization: adult humans display right frontal/left occipital asymmetries (Geschwind and Miller, 2001) forming an example of predictable, species-level cortical organization unique to humans that can be traced in the hominin lineage, as documented in the fossil record (see discussion below).

An important feature of cortical asymmetries is that they represent essentially a developmental phenomenon. Asymmetries can be observed in perisylvian regions and the planum temporale prenatally (30 gestational weeks; Chi et al., 1977a), and differences in gene expression between the two hemispheres are observed even earlier in the development (12–14 gestational weeks; Sun et al., 2005). During development, the right hemisphere may exhibit a faster tempo of development compared to the left hemisphere (Chi et al., 1977b; Sun et al., 2005) and the pattern of asymmetries seen in adults is either absent or reversed in infants and children. The typical adult-like pattern of asymmetry emerges during adolescence (Shaw et al., 2009). At the same time, structural asymmetries are either absent or reversed in several disorders – including dyslexia (Geschwind and Galaburda, 1985), autism, and developmental language disorder (Herbert et al., 2005). Changes in functional hemispheric dominance were reported in individuals with brain injuries (Joseph, 1986) and following corpus callosotomy (Gazzaniga et al., 1984). Taken together, these observations suggest that although development of asymmetries tends to be predictable in humans and may be primarily under genetic control, environment processing demands appear to influence the establishment of proper functional circuitry underlying functional lateralizations in humans.

Analyses of morphology of pyramidal neurons in cortical areas associated with lateralized behaviors suggest that the lateralization observed in gross anatomical studies find their equivalent at the cellular level. In language areas, the so-called “dendritic laterality” has been reported in Broca’s area, Wernicke’s area, and Rolandic motor areas (Scheibel et al., 1985; Jacobs and Scheibel, 1993). The Wernicke’s area equivalent in the right hemisphere was characterized by less neurophil, greater overlap among columns, and greater variability in orientation of pyramidal neurons. In the dominant (left) hemisphere, layer III pyramidal neurons were longer, more branched, and more spinous compared to the neurons in the right hemisphere. The hemispheric pattern changed with aging; in individuals older than 50 years, pyramidal neurons in the left hemisphere became more prone to degradation compared to the ones in the right hemisphere, resulting in the reversal of the dominance pattern. Unlike in younger individuals, the pyramidal cells in the left hemisphere of older individuals were shorter and less spinous than the cells in the right hemisphere (Jacobs and Scheibel, 1993). Pyramidal neurons in the language areas in the frontal lobe display a less clear pattern of hemispheric dominance. Scheibel et al. (1985) reported that the total dendritic length in Broca’s area was comparable to the length of dendrites in the homologous area on the right hemisphere; the same pattern holds for Rolandic areas. The differences, however, were noted at more subtle elements of neuronal structure: pyramidal neurons in the left hemisphere were more branched and displayed greater number of high-order segments, i.e., fourth, fifth, and sixth order segments from the cell body. In the right hemisphere, pyramidal neurons in both areas displayed more lower order segments (first, second, third order) compared to the neurons in the left hemisphere. The pattern was consistent in right-handed subjects, and the hemispheric specificities was reversed in left-handed subjects (Scheibel et al., 1985). The authors suggested that the observed pattern, namely different modification of segments relative to the proximity to the cell body, reflected segment-specific developmental timing.

The segments closer to the cell body are formed during development prior to the higher-order segments, thus before the emergence of complex, lateralized behaviors. The appearance of more branched higher order segments coincides with functional maturation of the left hemisphere as the dominant hemisphere. Alternatively, as the authors suggested, higher order segments may be more plastic, and greater branching of high order segments in the left hemisphere might represent a response to higher demands of the behaviors processed in the left hemisphere (Scheibel et al., 1985).

The study by Scheibel et al. (1985) highlights an important point in examining the variability of pyramidal neurons in humans: in their adult phenotype, pyramidal neurons reflect cell-autonomous influences, as well as computational responses imposed upon them based on the area they occupy. Different parts of a pyramidal neuron may not respond in the same way to environmental influences: the parts of pyramidal neurons maturing at the time of environmental input may be more responsive in modifying their morphology, while developmentally earlier parts may remain more stable.

### DEVELOPMENTAL PLASTICITY IN PYRAMIDAL NEURONS

The emergence of pyramidal neurons and their differentiation and establishment of proper synaptic connections represents the first step in the formation of cortical connectivity. In primates, cortical neurogenesis is limited to the first half of gestation. At embryonic day 40 (E40) in macaques and E43 in humans (Rakic, 1982), neuronal progenitor cells exit the cell cycle and migrate along radial glia toward their position in the developing cortical plate. Earlier born neurons are destined to occupy subgranular cortical layers (layers V/VI), whereas later born neurons migrate into supragranular layers (layers II/III; Rakic, 1982). In humans at 17 gestational weeks (gw), a set of neurons in the cortical plate starts displaying morphology typical of pyramidal neurons – large somata, three to five basal dendrites with developed secondary branches, and a distinct apical dendrite directed toward the marginal zone (Mrzljak et al., 1988). With the appearance of lamination in the cortical plate, it becomes possible to distinguish pyramidal neurons in the developing layer III from those in layer V: pyramidal cells in the developing supragranular layers appear less branched and less spinous compared to their layer V counterparts, displaying overall less mature morphology (Mrzljak et al., 1988). Despite being based on a small sample of prenatal human tissue, these studies show that already at this developmental stage layer III neurons are marked by variations – the neurons in the upper part of the layer III are less branched and shorter than their counterparts in the deeper portions of layer III (Marin-Padilla, 1970; Mrzljak et al., 1988). The differences in the morphology of pyramidal neurons based on their laminar affiliations will persist throughout development and into adulthood (Petanjek et al., 2008). Layer-specific developmental differences appear particularly prominent during the perinatal period, that is, the period marked by initial neuronal response to direct environmental stimuli (Bourgeois, 1997).

It is of particular interest that layer III pyramidal neurons in human PFC, i.e., the subset of neurons characterized by the most elaborate dendritic morphology and highest number of synaptic inputs in adulthood, are the least developed neurons at birth (Petanjek et al., 2008). The early post-natal period is marked by their extensive elaboration; by the end of the first year of life, layer III pyramidal neurons in PFC appear as developed as layer V pyramidal cells, and by the end of third year of life, they emerge as most complex neurons in the human cortex (Petanjek et al., 2008). The morphological development of pyramidal neurons tends to parallel cognitive maturation, with an increase in language abilities, working memory, and symbolic thought in human infants during the same period (Goldman-Rakic, 1987). Interestingly, further elaboration in the morphology of pyramidal neurons, although at a smaller scale, continues into adulthood (Petanjek et al., 2008), thus spanning the period of continued cognitive and behavioral maturation in humans. As environment plays a crucial role in establishing proper cortical circuitry, the immaturity of layer III pyramidal cells at birth, rapid modification in the first few post-natal years, coupled with a continued modification until adulthood, allows for establishment of basic circuitry while enabling further individuation (*sensu *Bourgeois, 2001), depending on individual experiences and the needs of a particular social environment.

Significant changes during the post-natal period in the developing amygdala suggest that environmental inputs play an important role in specifying its morphology. It has been demonstrated both in humans (Joseph, 1999) and macaques (Harlow and Harlow, 1969) that lack of interaction with conspecifics and the inability to form attachments during the first year of life results in social and emotional abnormalities that persist throughout adulthood, possibly underlined by improper initial inputs into the amygdala from the social surrounding of an infant. As an example, humans infants suffering from neglect soon after birth tend to develop severe emotional non-responsiveness and fear of strangers, whereas those deprived of care after 6 months of age display increased need for attention, but remain unable to develop proper social adhesion (Joseph, 1999). In macaques, changes in social behavior and increased anxiety in adults are related to early life stress such as maternal separation. In turn, neonatal amygdala dysfunction has been shown to underlie non-adaptive responses to environmental and social stimuli. This suggests that alterations in amygdala development are linked with external changes in the environment. Monkeys with neonatal lesions demonstrate increased fear behavior in social interactions compared to control monkeys (Thompson et al., 1969; Prather et al., 2001). In contrast, monkeys with lesions produced in adulthood engage in greater amounts of affiliative social interactions than controls, suggesting a lack of social fear (Emery et al., 2001).

Structurally, the amygdala primodium first appears during the embryonic period in humans as a thickening in the wall of the interventricular foramen at the time that the hemispheres begin to evaginate. It is contiguous with the hippocampus and closely related to the striatum. The amygdala nuclei form by the migration of neuroblasts from the germinal layer of the striatal ridge, or ventricular eminence (also referred to as ganglionic eminence, Humphrey, 1968; Ulfig et al., 2003; Muller and O’Rahilly, 2006). At first, three main subdivisions emerge: the anterior amygdaloid area, the corticomedial complex, and the basolateral complex. The anterior amygdaloid area is identifiable first, followed shortly by the corticomedial complex (the cortical, medial, and central nuclei) and then the basolateral complex. Before the end of the embryonic period fiber connections develop between the amygdaloid nuclei and the septal, hippocampal, and diencephalic regions (Muller and O’Rahilly, 2006).

In the fifth gestational month in humans, aggregations of cell columns extend from the ventricular eminence into the basolateral complex. The presence of radial glia (demonstrated by vimentin immunoreactivity) between the columns suggests that these aggregations represent early migratory systems. In the sixth and seventh gestational months the cell columns begin to lose their connections with the ventricular eminence and fibers are no longer found between the cell columns. Finally, in the eighth and ninth month the aggregates of cell columns are no longer present and the lateral nucleus appears distinctly separate from the ventricular eminence (Ulfig et al., 2003). In parallel with this development, punctate immunolabeling of GAP-43, which is correlated with synaptogenesis (McGuire et al., 1988), appears in the fifth gestational month in the corticomedial complex and in the seventh month in the basolateral complex. By the ninth month there is no longer evidence of GAP-43 in the amygdala (Ulfig et al., 2003).

The amygdala in primates is immature at birth and its development thus depends on incoming stimuli from the environment. Differentiation of individual amygdala nuclei continues from the embryonic period through the fetal period and on into the post-natal period. Many nuclei exhibit distinct developmental profiles. For example, post-natally in macaque monkeys, the nuclei of the basolateral complex demonstrate a dramatic enlargement in volume between birth and 3 months of age, with slower growth continuing beyond 1 year. In contrast, the medial nucleus is near adult size at birth, while the volume of the central nucleus is half the adult value at birth and exhibits slow but significant growth even after 1 year of age (Chareyron et al., 2012). At a cellular level, early pyramidal neurons can be distinguished in the human amygdala by the eighth and ninth gestational months. Similarly to the pyramidal neurons in the neocortex, these early pyramidal neurons are characterized by medium diameter dendrites that emerge from pyramidal-shaped soma, a stout branching dendrite emerging from opposite pole of the soma, and an axon emerging from the base of the pyramids. The onset of synaptogenesis is delayed in the basolateral complex relative to the corticomedial complex (Ulfig et al., 2003). Since the lateral nucleus is characterized as derived in its organization in humans (Barger et al., 2007, 2012) and functions as an important part of the network processing of social and emotional stimuli, it remains possible that a prolonged period of maturation enables establishment of social and emotional bonds extending beyond the mother; a feature in particular important in humans species, where sharing offspring care represents an evolutionary strategy for increasing reproductive success (Hrdy, 2005). Compared to humans, infant care is less extensively shared among group members in great apes and most Old World monkeys, and the nature of alloparenting thus differs between humans and other primates.

Among the Efé of Central Africa, for example, by 18 weeks of age infants spend more than half a day with caregivers other than their mothers, averaging about 14 caretakers including both related and unrelated individuals (Hrdy, 2005). In comparison, a systematic study of alloparental episodes among the chimpanzees in Mahale Mountains, Tanzania, suggests that only certain members of the troop (e.g., nulliparous females) tend to display interest into handling infants, whereas parous females remain indifferent to the offspring of other females (Nishida, 1983). A similar pattern was observed among Japanese macaques (*Macaca fuscata*; Hiraiwa, 1981). Even among the species where infant sharing is quite common, such as Barbary macaques (*M. sylvanus*; Small, 1990), the mother remains the primary caretaker of the infant, and alloparenting never reaches the extent seen in humans. Similarly, the development of ‘stranger distress’ is delayed in human infants compared to other primates, appearing at approximately 7 months in humans, 4 months in chimpanzees, and 3 months in macaques (reviewed in LaFreniere, 2005). Although the appearance of fear reaction to strangers doubtlessly depends on other cognitive (e.g., development of the concept of the caregiver; LaFreniere, 2005) and neural changes (e.g., neocortical maturation; Goldman-Rakic, 1987), developmental changes in the amygdala nevertheless underlie the emergent fear response in primates during the first year of life.

## EPIGENETIC AND MOLECULAR ASPECTS OF HUMAN BRAIN EVOLUTION

It has been proposed that the environment mediates the establishment of neuronal morphology by two mechanisms of plasticity: experience-expectant plasticity, preparing neuronal circuits for ubiquitous environmental inputs, and experience-dependent plasticity, responsive to the circumstances unique to each individual (Greenough et al., 1987). Experience-expectant plasticity likely reflects evolutionary mechanisms emphasizing a particular type of sensory processing shared by all members of a species (Greenough et al., 1987). This is manifested by overproduction of synapses during the perinatal period in cortical areas subserving the sensory system in question, followed by a rapid pruning of synapses at the end of the period. Experience-dependent plasticity, on the other hand, is less predictable, characterized either by prolonging the period of synapse overproduction or delaying the offset of synaptic pruning (Bourgeois, 1997). Synaptogenesis in the primate visual cortex represents a typical example of experience-expectant plasticity. In rhesus macaques, rapid production of synapses in primary visual cortex (V1) begins 2 months before term, becomes intensified around birth, and ends at post-natal day 61 (P61; Bourgeois, 1997). The rate of synapse production remains stable even if the monkeys are delivered before term – thus exposed to light prematurely compared to the full-term controls – although the maturation rate of synapses appears to proceed faster in pre-term macaques (Bourgeois et al., 1989). It has been proposed (Joseph, 1999) that development of the amygdala and associated cortical regions involved in processing emotional and social stimuli represent another example of experience-expectant maturation (Harlow and Harlow, 1969; Joseph, 1999).

Experience-expectant plasticity is often associated with critical periods in development (Greenough et al., 1987) and it is in particular prominent in the maturation of sensory systems. In contrast, the basic premise of experience-dependent plasticity proposes that the opportunity to acquire complex behaviors varies across individuals and that the nature of the acquired information will differ from one animal to the next (Greenough et al., 1987). This type of plasticity underlies acquisition of multifaceted behaviors, including navigating one’s social and ecological surroundings, language acquisition, and ability to acquire new technical and behavioral skills. Rather than providing a developmental window in which stimuli are necessary to establish functional circuitry, experience-dependent modifications are possible in late-maturing regions, depending on individual circumstances (Greenough et al., 1987). In macaques, rapid development of synapses proceeds uniformly in both V1 and PFC, although the two areas harbor two rudimentary different types of processing (Bourgeois et al., 1994). In humans, on the other hand, development of synaptic densities is postponed in PFC compared to other cortical regions (Huttenlocher and Dabholkar, 1997), suggesting that maturation of executive control in humans may be postponed compared to macaques, allowing for a prolonged period of modifications. Dendritic systems of pyramidal neurons in human PFC continue to mature longer than PFC neurons in macaques (Cupp and Uemura, 1980; Petanjek et al., 2008), with elaboration of dendritic branching continuing until adolescence (Petanjek et al., 2008) and maturation of spines proceeding until the third decade of life (Petanjek et al., 2011). The prolonged period of maturation of cortical microcircuitry in PFC thus encompasses two developmental stages unique to humans: childhood and adolescence (Bogin and Smith, 1996; Bogin, 1997). The additional period of cognitive plasticity in humans enables the acquisition of baseline skills necessary for successfully navigating social and ecological environments (Leigh and Park, 1998; Flinn, 2005), forming the basis for their elaboration in later life (Geary, 2005). It is important to note, however, that modifications in cortical microcircuitry continue throughout life, even without obvious pathologies or physical traumas (Jacobs and Scheibel, 2002), enabling modifications of behavioral responses to newly encountered circumstances.

A discussion about plasticity inevitably introduces the question of cell-intrinsic and epigenetic influences on the development, and the relative importance of each in influencing a particular aspect of neuronal morphology. The development of new comparative genomics, epigenetic analyses, and gene expression tools has catapulted interest in the molecular aspects of human brain evolution. Variability selection posits the importance of regulatory mechanisms of gene expression in lineages subjected to variability selection (Potts, 1998), with the activity especially prominent during development; comparative studies across primates have suggested differences in timing, increased importance of non-coding sequences, and accelerated rates of evolution of development-related genes in humans (Dorus et al., 2004; Prabhakar et al., 2006; Liu et al., 2012).

At the genomic level, several reported molecular events illustrate the complexity of human evolution. On one side, humans can acquire new genetic information. For example, KLK8 (also known as neuropsin) is a secreted-type serine protease that is involved in synaptogenesis, neurite outgrowth, and plasticity in the hippocampus and the neocortex (Mitsui et al., 1999). A human-specific point mutation gave rise to a novel functional isoform (type II) that is only expressed in humans during development in the embryo brain, suggesting a potential role in early CNS formation (Lu et al., 2007, 2009). On the other side, a loss of function is observed in the human genome, affecting a specific biochemical pathway. For example, the human deficiency of Neu5Gc is explained by the fixations of an inactivating mutation in the gene encoding CMP-*N*-acetylneuraminic acid hydroxylase, the rate-limiting enzyme in generating Neu5Gc in cells of other mammals. The mutation occurred after the split from our last common ancestor (Chou et al., 2002). Fixation in the ancestral population occurred at an unknown time thereafter and happens to be one of the first known genetic differences between humans and other hominids with an obvious biochemical readout. Together, these data are consistent with the presence of human-specific genomic alterations.

Alteration in gene expression is a common mode of evolutionary change and can result from multiple changes in the genome, affecting regulatory regions such as promoters and enhancers. These alterations may affect gene dosage, timing and localization. Some studies suggested several differences that seem human specific: the majority of genes showing expression differences between humans and chimpanzees are upregulated in the human cortex (Cáceres et al., 2003) and show a species-specific pattern of expression (Enard et al., 2002). Gene expressions in regions involved in complex cognitive tasks tend to resemble one another, differing from the expression profiles in primary processing areas (Khaitovich et al., 2004). At the same time, comparative studies of gene expression between humans and chimpanzees suggest that the overall pattern of gene activity during the post-natal period is shared between these two species. However, compared to chimpanzees, about half of genes specific to a particular developmental stage are expressed at different levels in humans. Moreover, the difference between the two species increases over time, with the greatest difference occurring at 10 years of age (Somel et al., 2009). Several functional groups of genes involved into synaptogenesis and neuronal function display prolonged expression in humans compared to chimpanzees and macaques; in humans, their levels remain high during the first 5 years of life whereas in chimpanzees their levels decline early in the post-natal period. As a comparison, the same set of genes is elevated prenatally in macaques (Liu et al., 2012). Overall, the comparative molecular analyses of brain development suggest a tendency toward heterochrony – with a prolonged period of expression in humans compared to other primates – an increased role of regulatory mechanisms, and regional differences in gene expression across distinct brain regions.

Throughout the life of an individual, the brain faces two opposable needs: on one side, maintenance of the established functional circuitry and on the other, remodeling of the circuits in response to newly imposed computational needs (Abrous et al., 2005). Different parts of the brain may have solved this dilemma differently: regions characterized by continuous neurogenesis (e.g., hippocampus) through the addition of new neurons and the establishment of new circuitry (van Praag et al., 2002), while the non-neurogenic regions (e.g., the neocortex) through modifications in morphology of the existing neurons (Abrous et al., 2005). Morphological changes of pyramidal neurons – length, branching, and the number and distribution of dendritic spines – have been reported in the cortex of human subjects following physical (Jacobs et al., 2003) and chemical (Glantz and Lewis, 2000) changes, or behavioral manipulations in laboratory animals (Bock et al., 2005; Cerqueira et al., 2007). In a study of macaques raised in a cage without enrichment and with only visual contact with conspecifics, Bryan and Riesen (1989) reported decrease in density of spines on apical dendrites in V1 pyramidal neurons, but no reduction in their overall branching complexity. The same conditions resulted in decreased length, arborization, and density of spines on apical dendrites in primary motor cortex (M1; Bryan and Riesen, 1989), suggesting that the effects of deprivation affected neurons in different cortical regions differently, and that some parts of pyramidal morphology (e.g., spines) appear more prone to environmental influence than the others. These findings tend to be supported by gene expression analyses: expression of the immediate early genes (IEGs) in the cortex has been associated with learning and memory (Kaufmann and Worley, 1999), and electrical activity in neurons appears to mediate the effects of brain-derived neurotrophic factors (BDNF) in the developing cortex (McAllister et al., 1996). Expression of some of IEGs seems to be focused specifically on dendrites (McAllister et al., 1996) and on dendritic spines (Schratt et al., 2006), facilitating rapid morphological modifications of the neurons.

An example of changes in neuronal morphology reported by Jacobs et al. (2003) suggests that the human cortex may respond to the same stressor differently than the cortex of other mammals. Several decades after undergoing corpus callosotomy, pyramidal neurons in layer III developed unusually long, branched, and spinous basal dendrites, which descended deep into subgranular layers. These “tap root’ dendrites were in particular common in Broca’s area (Jacobs et al., 2003), which shares connections with its homolog in the right hemisphere and receives numerous interhemispheric afferents from the right inferior temporal cortex (Di Virgilio and Clarke, 1997). The unusually developed basal dendrite, as the authors suggested, may represent an attempt by the neurons to maintain their function after losing cross-callosal inputs by increasing the area available for connections within the same hemisphere. In rabbits, callosotomy resulted in the decrease of spine number on oblique branches of apical dendrites in the parietal cortex, while at the same time the morphology of basal dendrites remained largely unaffected (Globus and Scheibel, 1967). These findings suggest that several factors – including the highly lateralized function of Broca’s area and an increased reliance on regulatory mechanisms modulating the relationship between cell structure and neuronal activity – may underline the observed differences in the modifications of neuronal morphology between the two species. The study thus reinforces conclusions implicit to numerous comparative studies – that the cortex of each species is a product of its evolutionary history, favoring a particular way of processing or, in morphological terms, a particular pattern of cortical connectivity that is layer-, area-, and likely species-specific. While it is reasonable to expect that the neurons with the same biophysical properties will respond to the stimulus in a similar way, regardless of the species or the area they occupy, functional demands imposed upon the neurons likely differ, and their morphology will change in response to the epigenetic factors differently, depending on nature of the network they form.

## THE DIRECT EVIDENCE OF HUMAN BRAIN EVOLUTION: THE FOSSIL RECORD

Fossil hominin endocasts can provide important clues to identify modifications of the human brain during evolution. An endocranial cast, or endocast, is a cast of the inner table of the cranial bones. Fossil endocasts are either naturally formed via filling and consolidation of sediment inside the braincase during the fossilization process, or artificially human-made. Endocasts of fossil specimens are the only available remnants of the morphology of their brains; as such, fossil hominin endocasts represent the only direct evidence of human brain evolution.

Endocasts preserve only some gross morphological characteristics of the brain’s outer surface, as pia mater, arachoid tisue, and dura mater form a buffer preventing the brain from leaving imprints in the inner cranium. Typically, estimates of cranial capacity can be reliably extrapolated based on the endocasts, whereas finer aspects of cerebral organization, such as gyral and sulcal pattern, remain more problematic and debatable (Holloway et al., 2004). Correlating microanatomical information with endocasts is a multistep process bridging microanatomy obtained from post-mortem histological sections with gross brain anatomy obtained from MRI. Such attempts have been made recently (e.g., Schenker et al., 2010; Annese, 2012; Yang et al., 2012), opening a promising field for future research. The second step is to evaluate the relationships between gross external neuroanatomy and endocranial morphology. Complex interactions throughout head ontogeny involve the brain, meninges, cranial vault, basicranium, face, mandible, and masticatory muscles (e.g., Moss and Young, 1960; Moss, 1968; Lieberman et al., 2000; Bastir et al., 2004; Bruner, 2004; Richtsmeier et al., 2006; Mitteroecker and Bookstein, 2008; Neubauer et al., 2009). Despite these interactions the shape of the cranial inner table (i.e., the shape of the endocast) reflects the shape of the brain until brain growth completion and throughout adulthood until incipience of brain tissue shrinkage (Courchesne et al., 2000; Resnick et al., 2003; Scahill et al., 2003; Kruggel, 2006; Sherwood et al., 2011; Ventrice, 2011). For this reason, endocranial volume and shape are used as proxies for brain size and shape.

The endocranial fossil record has been extensively reviewed (e.g., Bruner, 2003; Holloway et al., 2004; Falk, 2007, 2012). The ongoing study of the virtually reconstructed endocast of *Sahelanthropus tchadensis* (Brunet et al., 2002; Bienvenu et al., 2013), dated to 7 Ma (Mega Annum, a period of one million years) will open a unique window on the earliest stages of hominin brain evolution. Indeed, apart from this specimen, the earliest known hominin endocasts belong to australopiths dated around 3 Ma from South Africa and East Africa. They are formally separated into gracile (genus *Australopithecus*) and robust (genus *Paranthropus*) forms. Origins of the genus *Homo* are thought to be nested within genus *Australopithecus*, while robust australopiths are generally considered as side branches. The earliest *Homo* endocasts come from East Africa and date to less than 2 Ma. *Homo erectus sensu lato* is the earliest species known out of Africa around 1.8 Ma, found in Caucasus and Indonesia. *H. heidelbergensis* encompasses African and European fossils from the middle Pleistocene (between about 0.8 and 0.1 Ma). African *H. heidelbergensis* specimens may be ancestral to *H. sapiens*, while European specimens may be ancestral to *H. neanderthalensis*, Eurasian late archaic *Homo* ranging in age from about 0.2 Ma to 30,000 years ago. Australopiths are characterized by great ape-sized brains. When brain size began to increase in hominins is debated: increase in brain size began either gradually from around 3 Ma (Falk et al., 2000) or suddenly from around 2 Ma (Carlson et al., 2011; **Table 1**).

**Table 1:** Endocranial asymmetries in selected fossil hominins.

Specimen	Species	Age	Location	Petalias	Broca’s cap
Sterkfontein type 2	*Australopithecus africanus*	2.5 Ma	South Africa	No frontal petalia, occipital not preserved	Nascent?
MH1	*Australopithecus sediba*	2 Ma	South Africa	Right frontal	Nascent?
KNM-WT 17000	*Paranthropus aethiopicus*	2.5 Ma	East Africa	Right frontal-left occipital	Absent
OH 5	*Paranthropus boisei*	1.8 Ma	East Africa	Right frontal-left occipital?	Not preserved
SK 1585	*Paranthropus robustus*	1.5 Ma	South Africa	Left occipital	Absent
KNM-ER 1813	*Homo habilis*	1.8–1.9 Ma	East Africa	?^*^	Nascent?
KNM-ER 1470	*Homo rudolfensis*	1.8–1.9 Ma	East Africa	Pronounced right frontal-left occipital	Present
Any	Subsequent *Homo*	from 1.8 Ma	Africa, Eurasia	Pronounced right frontal-left occipital^**^	Present

*Sources: Holloway and de la Coste-Lareymondie (1982);, Holloway et al., (2004), Falk (2007), Grimaud-Hervé and Lordkipanidze (2010), Carlson et al. (2011), and Balzeau et al. (2012). *

* Not scored consistently throughout the literature.

** Most common pattern.

### EVOLUTION OF HUMAN BRAIN ONTOGENY

The evolution of hominin brain ontogeny is attracting increasing interest (Zollikofer and Ponce de León, 2010, 2013; Leigh, 2012; Neubauer and Hublin, 2012) and deserves special attention here. Ontogeny includes growth (increase in size with age) and development (modifications in shape with age). From the growth perspective, the brain of modern humans is already bigger at birth compared to newborn chimpanzees (400 versus 145 cc; Zollikofer and Ponce de León, 2013) and it experiences a growth spurt during the first two post-natal years. This rapid initial growth does not occur in chimpanzees (Sakai et al., 2013) and it may account for our large adult brains, three to four times bigger than the brains of chimpanzees (1350 versus 385 cc; Zollikofer and Ponce de León, 2013). Brain growth slows down after the growth spurt, and brain size approaches that of adults after eruption of the first molar. From the developmental perspective, endocasts of humans and chimpanzees already have distinct shapes at birth, reflecting different prenatal ontogenies: notably, human neonates have squared-off frontal lobes (Zollikofer and Ponce de León, 2013). During early post-natal development, the human brain undergoes an extensive period of growth and there are modifications of the endocranium, including expansion in the parietal area and widening of the post-erior temporal parts (Neubauer et al., 2010). This change results in a more globular shape of the human cranium compared to both chimpanzees and late archaic *Homo* (i.e., *H. heidelbergensis* and Neanderthals; Lieberman et al., 2002; Neubauer et al., 2010; Gunz et al., 2012, but see also Ponce de León et al., 2013 for shared patterns among hominids). Although each extant ape species evolved its own ontogenetic trajectory, as exemplified by the differences between chimpanzees and bonobos (Lieberman et al., 2007; Durrleman et al., 2012), the early post-natal growth spurt and the associated “globularization phase” appear to be developmental features unique to anatomically modern humans and are either absent, or undetectable, in the developing great ape crania.

An important topic in paleoneurological studies is dating the transition from a more ape-like pattern of brain growth and development to a modern human pattern. There is some support for the idea that fossil hominin maternal pelvic dimensions can be used as an indirect source of information for neonatal brain size as in modern humans (Tague and Lovejoy, 1986), but it has also been argued that australopith female pelvic dimensions are larger than neonatal neurocranial dimensions, and obstetrical constraints were absent in australopiths as in extant great apes (Leutenegger, 1987). Moreover, taxonomic attribution of some important pelvic remains is also debated (Simpson et al., 2008; Ruff, 2010). For these reasons, we will only review the evidence coming directly from the endocasts of juvenile fossil hominins, in a chronological order.

Australopith brain ontogeny is documented mainly by the endocasts from Dikika and Taung. The Dikika child (*Australopithecus afarensis*), dated to 3.3 Ma, has an estimated age at death of approximately 3 years and an estimated endocranial volume between 275 and 330 cc (Alemseged et al., 2006). The Taung child (*A. africanus*; Dart, 1925), dated to 2.6–2.8 Ma (McKee, 1993), has an estimated age at death between 3.5 and 4 years (Lacruz et al., 2005) and an estimated endocranial volume of 405 cc (Neubauer et al., 2012). Brain ontogeny in early *H. erectus* is documented by one specimen, the 1-year-old Mojokerto child, dated to 1.8 Ma and with an estimated endocranial volume of 663 cc (Coqueugniot et al., 2004). In *H. neanderthalensis*, one specimen of special interest is the 1 to 2-week-old infant from Mezmaiskaya, Russia (Golovanova et al., 1999), dated to 0.073–0.063 Ma, with an endocranial volume estimated between 414 and 436 cc (Ponce de León et al., 2008; Gunz et al., 2012). *H. neanderthalensis* is probably the best known fossil hominin species concerning brain ontogeny, the whole range of individual ages being sampled, from the neonate of Mezmaiskaya to the “old man” of La Chapelle-aux-Saints.

**FIGURE 2:**
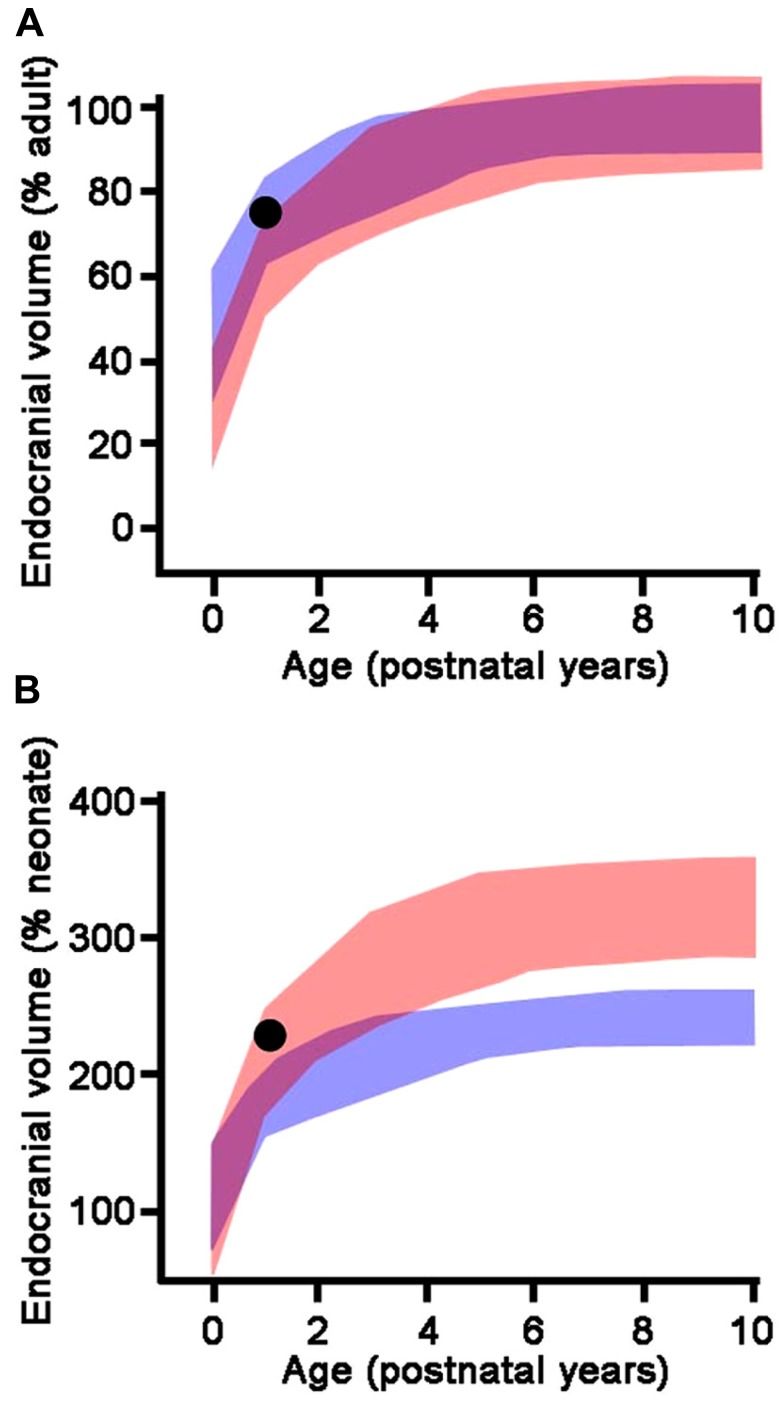
**Endocranial growth trajectories as a proportion of adult endocranial volume (A) and neonatal endocranial volume (B).** Red areas represent modern human growth trajectories (mean ± 1 standard deviation). Blue areas represent growth trajectories for chimpanzees (mean ± 1 standard deviation). Purple areas represent overlap between human and chimpanzee growth trajectories. Black dot: *Homo erectus* (Mojokerto) infant dated at 1.8 Ma (average values for estimated age, expected adult endocranial volume, and predicted neonatal endocranial volume). As a percentage of its expected adult endocranial volume, the *Homo erectus* child follows a growth trajectory similar to chimpanzees, while as a percentage of its predicted neonatal endocranial volume, he falls within the modern human range of variation. This particular pattern accounts for the lower endocranial volume of *Homo erectus* compared to modern humans (high percentage of adult endocranial volume reached early in ontogeny), associated with an early postnatal brain growth spurt. Adapted from Zollikofer and Ponce de León (2010).

The endocranial volume of a juvenile fossil can be compared to the endocranial volume of humans and apes of the same age in absolute terms, as a proportion of the estimated adult brain size, or as a proportion of the estimated neonatal brain size (Zollikofer and Ponce de León, 2010). For a fossil hominin species, estimated adult brain size is calculated as the average of the endocranial volumes of the conspecific adult specimens of the same sex. Estimated neonatal brain size is predicted from the regression of adult brain size versus neonate brain size in extant anthropoids (DeSilva and Lesnik, 2008). These three modes of comparison (absolute brain size, percentage of adult brain size, percentage of neonate brain size) may lead to different conclusions (**Figure 2**). Absolute brain growth curve and growth trajectory expressed as a percentage of neonatal brain size prove to be more discriminatory and reveal whether a species experiences a brain growth spurt or not, independently from adult brain size.

The Dikika endocast has the expected volume for a chimpanzee of the same age. The average estimated endocranial volume for adult female *A. afarensi*s is 375–425 cc (Alemseged et al., 2006). The endocranial volume of the Dikika child expressed as a percentage of this expected adult endocranial volume is in the overlapping ranges of chimpanzees, gorillas, and humans. As a proportion of its estimated neonatal brain size, the Dikika endocast falls within the variability range of chimpanzees (Zollikofer and Ponce de León, 2013). The Taung child is within the chimpanzee range of variation concerning the percentage of adult endocranial volume and neonatal endocranial volume (Zollikofer and Ponce de León, 2013). However, its absolute endocranial volume is slightly greater than expected for a chimpanzee of similar age (Zollikofer and Ponce de León, 2013). Estimates of australopith neonate brain size are slightly larger than for chimpanzees (180 cc versus 150 cc; DeSilva and Lesnik, 2008), implying that chimpanzees and australopiths displayed different prenatal growths. The partially fused metopic suture observed in the Taung endocast highlights this potential difference with chimpanzees (Falk et al., 2012). The Taung metopic suture may be correlated with an enlarged neonate brain size, rapid early post-natal brain growth, and squaring-off of the frontal lobes.

With *H. erectus*, the ontogenetic trajectory approaches the one for modern humans. The Mojokerto child has an estimated endocranial volume which falls at the lower end of the modern human range (Zollikofer and Ponce de León, 2013). The average adult endocranial volume in *H. erectus* is lower than in modern humans; consequently, the Mojokerto child has reached a high proportion of its expected adult brain size as is the case in chimpanzees (**Figure 2A**), which led Coqueugniot and colleagues (2004) to the conclusion that the growth pattern of *H. erectus* was similar to that of chimpanzees. However, the estimated neonatal brain size of *H. erectus* is clearly larger than that of chimpanzees, probably about twice as large (Leigh, 2006; DeSilva and Lesnik, 2008; Zollikofer and Ponce de León, 2013). When expressed as a percentage of the estimated neonatal endocranial volume, which yields better discrimination among taxa (Zollikofer and Ponce de León, 2010), the Mojokerto child falls well within the modern human range and out of the chimpanzee range (**Figure 2B**). From this, it appears that *H. erectus* experienced an early post-natal brain growth spurt, although for a shorter period than modern humans, which led to smaller adult brain sizes.

As evidenced by the Mezmaiskaya specimen, the neonate endocranial volume in Neanderthals was similar to modern humans, around 400 cc (Hüppi et al., 1998; Ponce de León et al., 2008; but see Coqueugniot and Hublin, 2012). The pattern of brain growth as a proportion of adult endocranial volume is similar in *H. neanderthalensis* and modern humans. As *H. neanderthalensis* reach a higher adult endocranial volume than modern humans, they express differences in absolute brain growth and in the pattern of brain growth as a percentage of neonate endocranial volume. Higher values are reached because of a more sustained post-natal brain growth spurt. The growth pattern of *H. neanderthalensis* may indeed be similar to that for ancient fossil *H. sapiens*, as a decrease in brain size has been reported in modern humans since about 0.03 Ma (Henneberg, 1998). While *H. neanderthalensis* and *H. sapiens* have similar endocranial shapes at birth (Gunz et al., 2012; but see Ponce de León et al., 2008; Zollikofer and Ponce de León, 2013), their adult endocasts have different shapes, and a recent study suggested differences in their brain organization (Pearce et al., 2013). Each species appears to reach similar brain size via distinct developmental pathways: the globularization phase occurring during the brain growth spurt is an autapomorphy (uniquely derived character state) of *H. sapiens* absent in Neanderthals (Lieberman et al., 2002; Gunz et al., 2012), which retain a similar developmental pattern to *H. erectus* (Bruner et al., 2003; but see also Ponce de León et al., 2013 for patterns present in great apes). Overall, the fossil record of juvenile endocasts suggests that the modern human brain growth pattern became established gradually from about 2 Ma in genus *Homo* (growth spurt), or even already in australopiths between 2 and 3 Ma (larger neonatal brain size). Conversely, the globularization phase typical of modern human brain development has so far not been established in the archaic *Homo.*

As discussed earlier, human cerebral hemispheres are highly specialized for different types of information processing (Gazzaniga, 2000), and this functional lateralization has its structural correlates at a gross level. Petalias, the differential expansion of one of the frontal or occipital lobe compared to its contralateral homologous, leave an impression and can be traced on the inner surface of the cranium. Fronto-occipital petalias occur together with a distortion of the midsagittal plane known as Yakovlevian torque, in which right frontal and left occipital lobe protrude across the midline, changing the position of the interhemispheric fissure (Toga and Thompson, 2003). Most pre-adolescent humans are characterized by a left frontal-right occipital petalial pattern (Ventrice, 2011), which reverses at adolescence, so that the most widespread adult human pattern is an association of a right frontal petalia and left occipital petalia (LeMay, 1976), in correlation with right-handedness (Galaburda et al., 1978). This pattern is also dominant in great apes, but to a lesser degree (Balzeau and Gilissen, 2010; Balzeau et al., 2012). No australopith petalial pattern approaches the pronounced right frontal-left occipital petalias observed in modern humans. Such marked petalias appear in early *Homo *around 1.8–1.9 Ma ago (**Table 1**). Taken together, the insights from the fossil endocasts suggest that structural lateralization typical of our species first appeared with the emergence of the earliest *Homo*. The petalias observed in fossil *Homo* may reflect the emphasis on preferential processing of certain tasks in one hemisphere over another, supporting the view that cerebra of the early members of our genus, in addition to an increase in size, were characterized by changes in organization and in the patterns of information processing compared to australopiths.

## CHALLENGES FOR THE FUTURE

**FIGURE 3:**
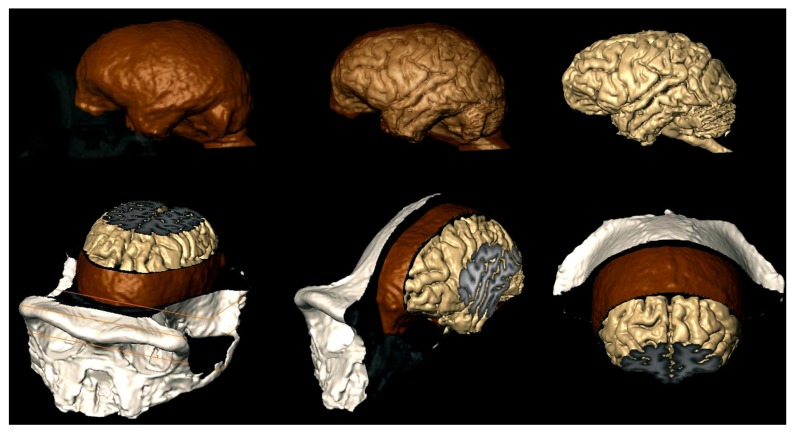
**MRI techniques used to study the brains of chimpanzees (Semendeferi et al., 2002), can now investigate the relationship between the brain and the endocast as shown here.** Brain is beige; endocast is brown; exocranium is white. Top row: exocranium (left) and endocast (middle) are shown transparent. Bottom row: MRI slices reveal internal structures of the brain, meninges, and bone.

Bringing together information on the structure of the human brain, its evolution, and development from endocasts through neural systems, neuronal morphology, and epigenetic control of cortical development is a multistep task. It involves the study of the relationship between endocranial morphology and gross external neuroanatomy (**Figure 3**), as well as the relationship between gross external neuroanatomy and microanatomy (**Figure 1**). This task also goes beyond developmental influences on the establishment of adult morphology and encompasses instead the full spectrum of the human condition, including aging, cortical modifications in cognitive and neurodegenerative disorders, and comparison with closely related species. With respect to the fossil record, analyses of endocast to brain relationships remain scarce (Connolly, 1950; Fournier et al., 2011; Ventrice, 2011). From a methodological point of view, more of such studies are needed, as they are crucial in forming inferences about brain anatomy of fossil hominids based from the imprints they left on the endocranium. Notably in the context of brain aging the brain tissue shrinks from adolescence onward in humans, while the volume occupied by cerebrospinal fluid and ventricles increases (Courchesne et al., 2000; Wanifuchi et al., 2002; Resnick et al., 2003; Scahill et al., 2003; Kruggel, 2006; Sherwood et al., 2011; Ventrice, 2011). Endocranial volume reaches a plateau at brain growth completion and, contrary to the brain, it is not significantly modified with aging (Courchesne et al., 2000; Scahill et al., 2003; Kruggel, 2006; but see Royle et al., 2013). It is reasonable to assume that neural tissue shrinkage within the solid, non-shrinking neurocranium, results in an increased gap between the brain and its case, filled with cerebrospinal fluid. This increase in the distance between the pial and endocranial surface with aging may explain why the endocranial impressions left by the growing brain become smoother in aging human individuals (Connolly, 1950; Grgurević et al., 2004; Zollikofer and Ponce de León, 2013). In addition, aged brain shrinkage is accompanied by a thickening of the inner cranial table (Royle et al., 2013), likely resulting in the osteoblastic filling of the endocranial gyral impressions (Tobias, 2006). The brain does not shrink significantly in aging chimpanzees (Sherwood et al., 2011) or in rhesus monkeys (Herndon et al., 1998), except in the most geriatric specimens (Herndon et al., 1999; Shamy et al., 2011). The smoothing of endocranial imprints from young adulthood in apes (Connolly, 1950) is more likely due to the continued expansion of the endocranial cavity after the completion of brain growth (Zollikofer and Ponce de León, 2013). The increased magnitude of brain shrinkage in humans may be a consequence of an extended lifespan (Sherwood et al., 2011) as increased longevity is a recent acquisition of modern humans (Caspari and Lee, 2004; Trinkaus, 2011). In this context, a study of the correlation between the level of endocranial gyral and sulcal details and age across hominin species would enable us to assess whether brain shrinkage only occurs in modern humans, or also happened in extinct human species with shorter lifespans.

Beyond endocasts, the study of the relationship between gross external neuroanatomy and microanatomy of the brain tissue is of special importance to the field of human neuroscience as a whole (e.g., Amunts et al., 1999; Schenker et al., 2010; Annese, 2012; Yang et al., 2012), and we expect that as such information becomes increasingly available, it will also assist in the meaningful interpretation of hominin endocasts in the years to come. Bridging different levels of analysis is a challenge and one good example of the types of complexities involved is provided by attempts to reconstruct the evolution of Broca’s area.

Broca’s area is defined cytoarchitectonically as the combination of Brodmann’s areas (BA) 44 and 45. Macroanatomically, Broca’s area roughly corresponds to a region in the inferior frontal lobe including the pars opercularis and the pars triangularis, bounded by specific sulci. However, the correspondence between sulcal pattern and cytoarchitectonic areas is loose in humans (Amunts et al., 1999). Broca’s area is larger on the left hemisphere than its contralateral homologous area in modern humans, according to both macroanatomical MRI-based studies (Foundas et al., 1998) and histological analyses (Uylings et al., 2006). These asymmetries are reflected in human endocasts, and lateralizations in the anterior language area were traditionally scored based on the appearance of Broca’s cap, i.e., the lateral and inferior bulging on the third inferior frontal convolution on the left hemisphere which corresponds to the anterior portions of Broca’s area (BA 45 and BA 47; Falk, 1987; Holloway et al., 2004). The presence of the asymmetries is typically determined by comparing the measurements for width of the left and the right frontal lobe measured at the level of the cap. Even subtle differences in the measurements, coupled with qualitative observations, are indicative of differences in the extent of Broca’s cap between the hemispheres (e.g., Broadfield et al., 2001). Broca’s cap appears in early *Homo* around 1.8–1.9 Ma ago (**Table 1**) and great ape and australopith endocasts do not have a Broca’s cap as modern humans do (Falk, 1987). Even though Broca’s cap is absent in apes, an MRI-based quantification of the macroanatomical features of Broca’s area homolog in African ape brains shows a significant leftward asymmetry based on ape typical sulcal patterns for the inferior frontal lobe (Cantalupo and Hopkins, 2001; but see also Sherwood et al., 2003b). At the same time, even though Broca’s area can be cytoarchitectonically defined in both humans and chimpanzees (Schenker et al., 2008), cytoarchitectonic asymmetry appears to be uniquely human (Schenker et al., 2010), suggesting that the insights gained from the three levels of evidence – endocasts, soft tissue analyses, and cytoarchitectonics – are still in need of better integration. Future studies should investigate possible asymmetries in the morphology of pyramidal neurons between the two hemispheres in additional species in primates, and ultimately asymmetric expression of genes. As discussed previously (Scheibel et al., 1985), the differences in dendritic morphology of pyramidal neurons between two hemispheres are often subtle and it remains to be seen whether morphological analysis of neurons in other hominids will shed additional light at the discrepancy between macroscopic (Cantalupo and Hopkins, 2001) and cytoarchitectonic (Schenker et al., 2010) findings. Moreover, a major challenge will be to disentangle the functional attributes of these different structural levels. Finally, a comprehensive understanding of Broca’s area structure and function also needs increased sample sizes, boundaries of regions of interest consistently defined across levels to allow comparisons among different studies, and developmental insights.

Reconstructing the evolutionary emergence of the neurobiological phenotype that underlies the unique human cognitive and behavioral specializations in development and adulthood is a multistep, multifield endeavor that requires contributions from molecular, neuroanatomical, and paleontological perspectives. Although some of our focus here has been on neocortical pyramidal neurons, we attempted to demonstrate how the insights gained from different fields can be combined to construct an evolutionary history of the human brain at several levels. We focused specifically on three aspects of human brain anatomy – asymmetries, development, and age-related changes – as those provide a fertile ground for combining different perspectives in creating testable scenarios about human brain evolution. Compared to other primates, the human brain displays specificities in the morphology of excitatory neurons in the neocortex, differences in macroscopic organization, unique patterns of post-natal development, and responds to the same environmental influences differently compared to the brains of other mammals. All of these features may have been facilitated by an expanded period for establishing cortical circuitry in humans. At the same time, rapid modifications can be achieved throughout lifetime, thus providing a neural substrate for behavioral and cognitive capacities unique to our species.

Over recent decades, the number of fossil specimens has greatly expanded, and so has our knowledge of the genetic and molecular variations across primates. Long-term studies in the field have yielded additional insights into behavioral variations, adaptations, and cognitive potentials of non-human primates. The analyses of post-mortem brain material have begun to examine variation across primates – including the great apes – focusing on the organization of the brain typical of each species in the context of its behavioral, ecological, and cognitive adaptations. To understand the evolutionary history of the human brain, human behavioral specificities and the neural circuitry enabling their appearance must be placed within the larger context of similar behaviors and structures in other primates. At the same time, these characteristics must also be placed within the context of other human adaptations, exemplified by social and cognitive aspects unique to our species. While it is challenging to fully integrate the three lines of evidence discussed in this paper into a comprehensive analysis of human brain evolution, we hope to have opened a discussion across disciplines and to have provided opportunities for further studies surpassing the limitations of each individual field.

## Conflict of Interest Statement

The authors declare that the research was conducted in the absence of any commercial or financial relationships that could be construed as a potential conflict of interest.
